# Monogenic Familial Chylomicronemia Syndrome in Children: Clinical Divergences and Management Paradigms

**DOI:** 10.7759/cureus.114010

**Published:** 2026-08-05

**Authors:** Sin Yi Cindy Chan, Ho Chung Yau, Ka Yee Chan, Antony Fu

**Affiliations:** 1 Department of Paediatrics and Adolescent Medicine, Princess Margaret Hospital, Hong Kong, HKG; 2 Department of Paediatrics, Prince of Wales Hospital, Hong Kong, HKG; 3 Department of Paediatrics, The Chinese University of Hong Kong, Hong Kong, HKG

**Keywords:** children, familial chylomicronemia syndrome, gpihbp1 mutation, hypertriglyceridemia, lpl deficiency

## Abstract

Monogenic familial chylomicronemia syndrome (FCS) is a rare autosomal recessive disorder caused by disrupted intravascular lipoprotein lipase (LPL) pathways. Phenotypic variation often leads to diagnostic delays. We contrast two unique pediatric presentations of FCS, highlighting distinct pathophysiology. Case 1 describes a 21-month-old girl with eruptive xanthomas, which was initially misdiagnosed as an infectious rash, later revealed to be secondary to severe hypertriglyceridemia of 100.6 mmol/L (reference range < 0.8 mmol/L). Molecular testing confirmed a genetic mutation in the GPIHBP1 gene. Case 2 describes a 2-day-old neonate with incidental finding of lipemia during jaundice evaluation, subsequently revealing a fasting triglyceride level of 8.5 mmol/L. Genetic analysis identified compound heterozygous mutations in the LPL gene. Case 1 required intensive insulin therapy, strict dietary fat restriction and adjunctive gemfibrozil. Case 2 was managed via dietary restriction and medium-chain triglyceride formula supplementation. This two-case series illustrates the diagnostic challenges of FCS in pediatric practice and the contribution of molecular confirmation to establishing the underlying aetiology and informing counselling and long-term management.

## Introduction

Familial chylomicronemia syndrome (FCS) is an ultra-rare autosomal recessive disorder of intravascular triglyceride hydrolysis, affecting approximately 1-10 individuals per million [1]. It results from biallelic pathogenic variants that abolish lipoprotein lipase (LPL)-mediated lipolysis, most often in LPL itself and less frequently in the genes encoding its cofactors and interacting proteins, including apolipoproteins (APOC2, APOA5), the molecular chaperone lipase maturation factor 1 (LMF1), and glycosylphosphatidylinositol-anchored high-density lipoprotein-binding protein 1 (GPIHBP1), the endothelial protein that binds LPL and shuttles it to the capillary lumen [1,2].

Presentation is heterogeneous, ranging from eruptive xanthomas, lipemia retinalis, and hepatosplenomegaly to life-threatening acute pancreatitis, and features may be absent or nonspecific in early life; diagnosis is consequently often delayed at a reported median age of 24 years [3]. Clinical scoring systems such as the North American FCS (NAFCS) Score offer objective diagnostic criteria, incorporating persistently elevated triglycerides (>10 mmol/L, 880 mg/dL) refractory to conventional lipid-lowering therapy, a triglyceride-to-total-cholesterol ratio >8 (in mg/dL), apolipoprotein B below 1.0 g/L, current age, age at onset, body mass index, pancreatitis history, and secondary contributors to hypertriglyceridemia. A score ≥60 indicates definite FCS with 100% specificity and 67% sensitivity [3]. Diagnosis should nonetheless be confirmed genetically whenever possible, although causal biallelic variants are identified in only approximately 77% of clinically defined cases [1].

Genotype-phenotype correlation remains poorly defined. Age at presentation, presenting features, and severity vary considerably between patients, yet this variability does not track with the causative gene: the molecular subtypes are largely similar phenotypically [2], and marked differences are seen even among individuals sharing a genotype. Neither the causative gene nor the clinical course can therefore be predicted from the other.

Management remains lifelong dietary fat restriction to 10-15% of total energy intake, supplemented with medium-chain triglycerides and fat-soluble vitamins [4]. Novel therapies such as apolipoprotein C-III-directed agents have transformed adult treatment but are not licensed in children [5].

In this case series, we report two children with genetically confirmed FCS arising from defects at different points in the LPL pathway: a 21-month-old toddler with a homozygous GPIHBP1 variant whose diagnosis was delayed by atypical cutaneous findings, and a neonate with compound heterozygous LPL variants identified incidentally through visibly lipemic blood. These cases illustrate the diagnostic difficulty of FCS in pediatric practice and the role of early molecular confirmation in directing family counseling and individualized management.

## Case presentation

Case 1

**Figure 1:**
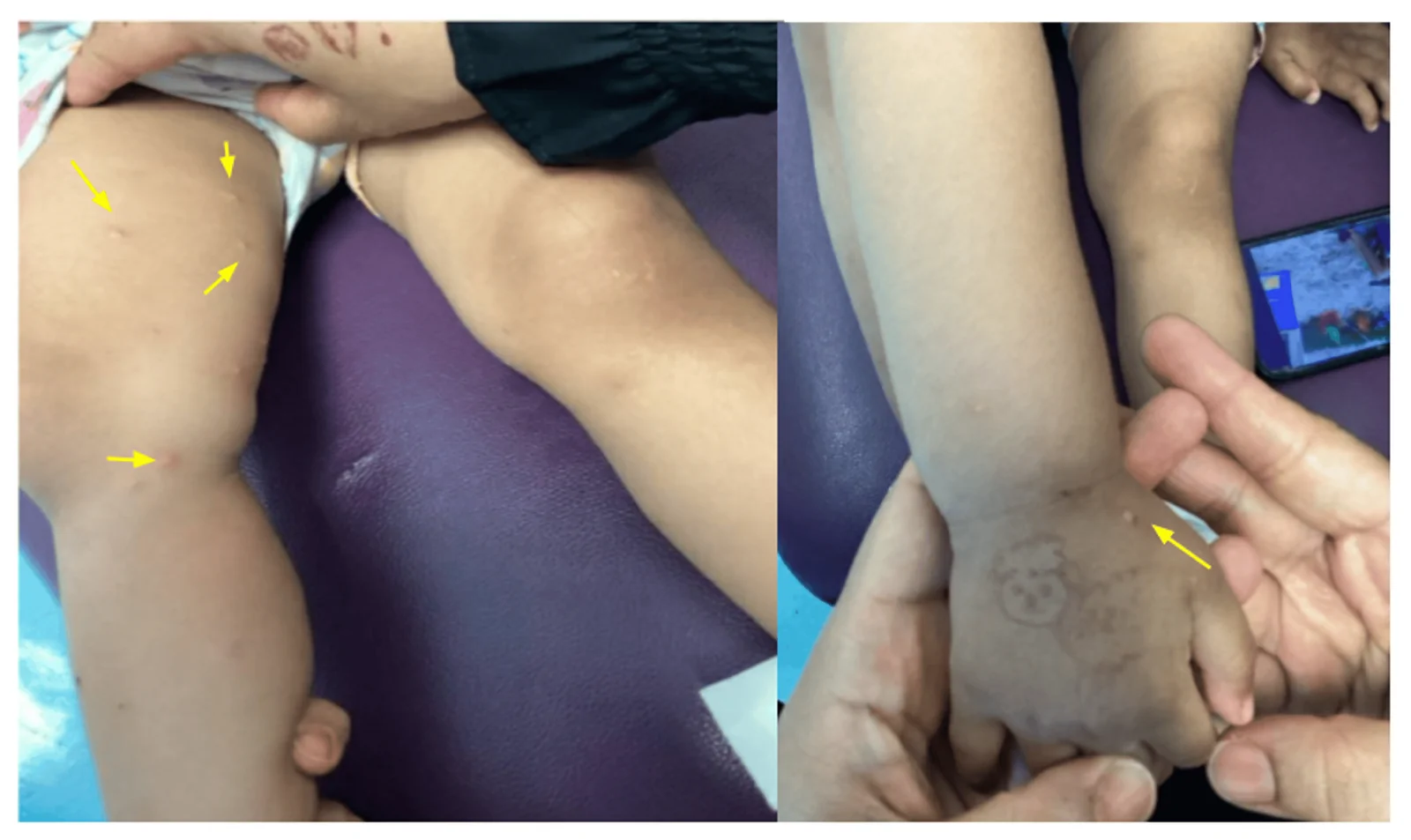
Clinical skin manifestations of the 21-month-old female patient (Case 1). Multiple discrete, non-confluent vesiculopapular lesions distributed over the trunk, lower limbs, and dorsal aspects of the hand, mimicking an infectious papulovesicular exanthem but representing atypical eruptive xanthomas (as indicated by yellow arrows).

A 21-month-old Pakistani girl initially presented a diagnostic conundrum with a one-year history of generalized, treatment-resistant papulovesicular rash. Born at term to consanguineous parents, she had otherwise been thriving with normal growth and development. Physical examination revealed multiple discrete vesiculopapular lesions distributed over her trunk, limbs, and groin, notably sparing the palms and soles (Figure 1). Given her age and the lesion morphology, infectious etiologies were primarily suspected, while classical lipid stigmata - such as eruptive xanthomas or xanthelasma - were not initially recognized.

**Figure 2:**
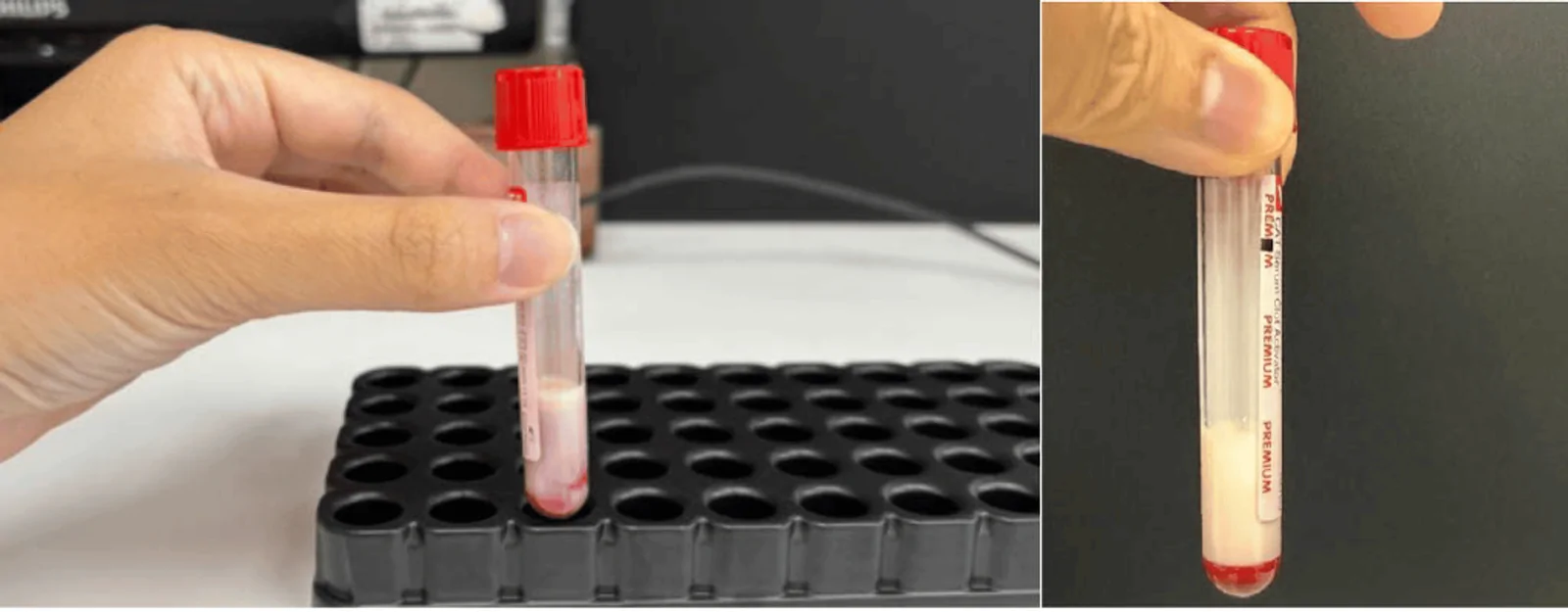
Visual presentation of a routine centrifuged whole blood sample from Case 1. The specimen demonstrates profound macroscopic lipemia, exhibiting a characteristically dense, thick milky supernatant layer resembling strawberry cream.

She was admitted for further infectious disease evaluation. However, the diagnostic turning point came not from an advanced microbiological assay, but from a simple visual observation of her routine blood draws. Repeated samples were flagged by the laboratory for being profoundly lipemic - a finding initially dismissed as a post-prandial artefact. The key diagnostic step occurred when the clinical team visually inspected the spun serum tube, which revealed a thick, milky supernatant resembling strawberry cream (Figure 2).

This stark visual clue immediately shifted the diagnostic paradigm, prompting a targeted lipid profile. The results confirmed a markedly elevated triglyceride level of 100.6 mmol/L and total cholesterol of 14.2 mmol/L, leading to the realization that the mysterious "infectious" lesions were, in fact, atypical eruptive xanthomas. Detailed laboratory results are shown below (Table 1).

**Table 1:** Biochemical investigations at diagnosis for case 1 Apo: apolipoprotein; HbA1c: glycated haemoglobin; HDL: high-density lipoprotein; LDL: low-density lipoprotein; —: not applicable / not reported. Lipoprotein(a) < 75 nmol/L is considered to exclude significant lipoprotein(a)-related cardiovascular risk.

Investigation	Result	Reference range
Lipid profile		
Total cholesterol	14.3 mmol/L	< 4.4 mmol/L
Triglyceride	100.6 mmol/L	< 0.8 mmol/L
LDL-cholesterol (direct)	Cancelled due to markedly elevated triglycerides	—
HDL-cholesterol	Cancelled due to markedly elevated triglycerides	—
Lipoprotein assay		
Lipoprotein(a)	19 nmol/L	< 75 nmol/L
Apolipoprotein A1	0.78 g/L	0.92–1.83 g/L
Apolipoprotein B	0.44 g/L	0.40–0.90 g/L
Apo B : Apo A1 ratio	0.6	< 0.8
Lipoprotein pattern	Dense chylomicron band detected	—
Other investigations		
Serum amylase	8 U/L	29–118 U/L
Serum lipase	18 U/L	< 32 U/L
HbA1c	4.80%	< 6.5%

**Figure 3:**
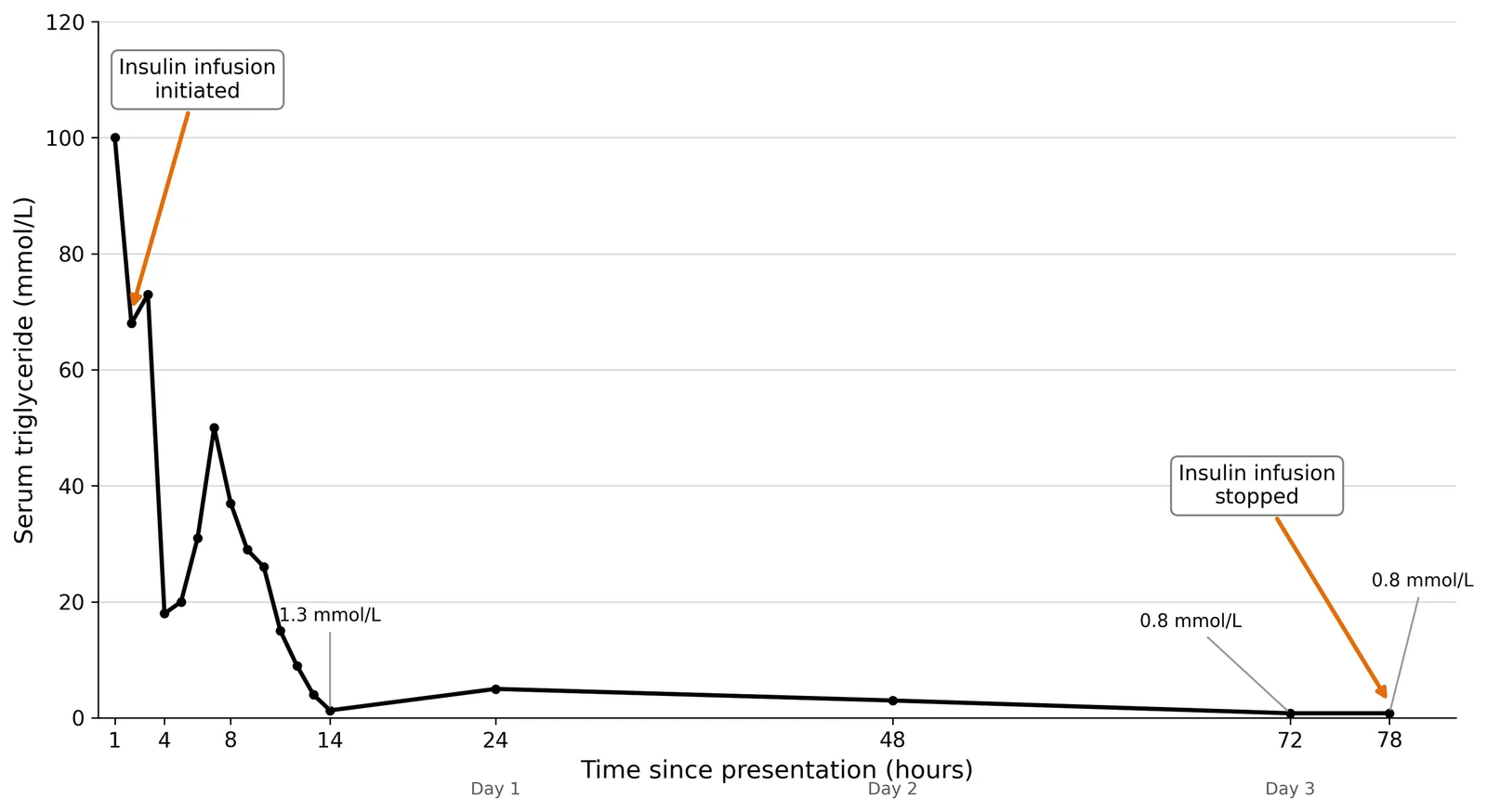
Longitudinal biochemical trend lines demonstrating serum triglyceride (TG) levels in mmol/L over hours following the clinical initiation of a continuous intravenous insulin infusion in the pediatric intensive care unit (Case 1). Rapid clearance of chylomicrons is achieved within 14 hours.

Due to profound hypertriglyceridemia, the patient was admitted to the pediatric intensive care unit, placed on nil per os (NPO) status, and started on intravenous insulin infusion aiming to accelerate triglyceride clearance and preempt complications from severe hypertriglyceridemia. The dosage of insulin infusion was titrated between 0.05 and 0.1 units/kg/hour, with concurrent intravenous dextrose infusion with a glucose infusion rate (GIR) of approximately 11 mg/kg/min to maintain euglycemia. Glucose and serum electrolytes - particularly potassium - were measured every 1-2 hours during insulin infusion. No adverse events occurred during treatment. The insulin infusion was continued until serum triglyceride level fell below 5.6 mmol/L - the threshold below which the risk of triglyceride-induced pancreatitis is considered low; insulin infusion was then tapered and discontinued. Serum triglycerides decreased from >100 mmol/L to 1.3 mmol/L within 14 hours of initiation. A transient rebound in triglyceride level to 6 mmol/L occurred at 24 hours, which was subsequently normalised to 0.8 mmol/L by 72 hours (Figure 3). Serial pancreatic enzymes, abdominal computed tomography, and fundoscopy revealed no evidence of pancreatitis or lipemia retinalis. Insulin infusion was gradually tapered and ceased after three days at 78 hours, and a strict very-low-fat diet of less than 8% of total daily energy intake was initiated.

**Figure 4:**
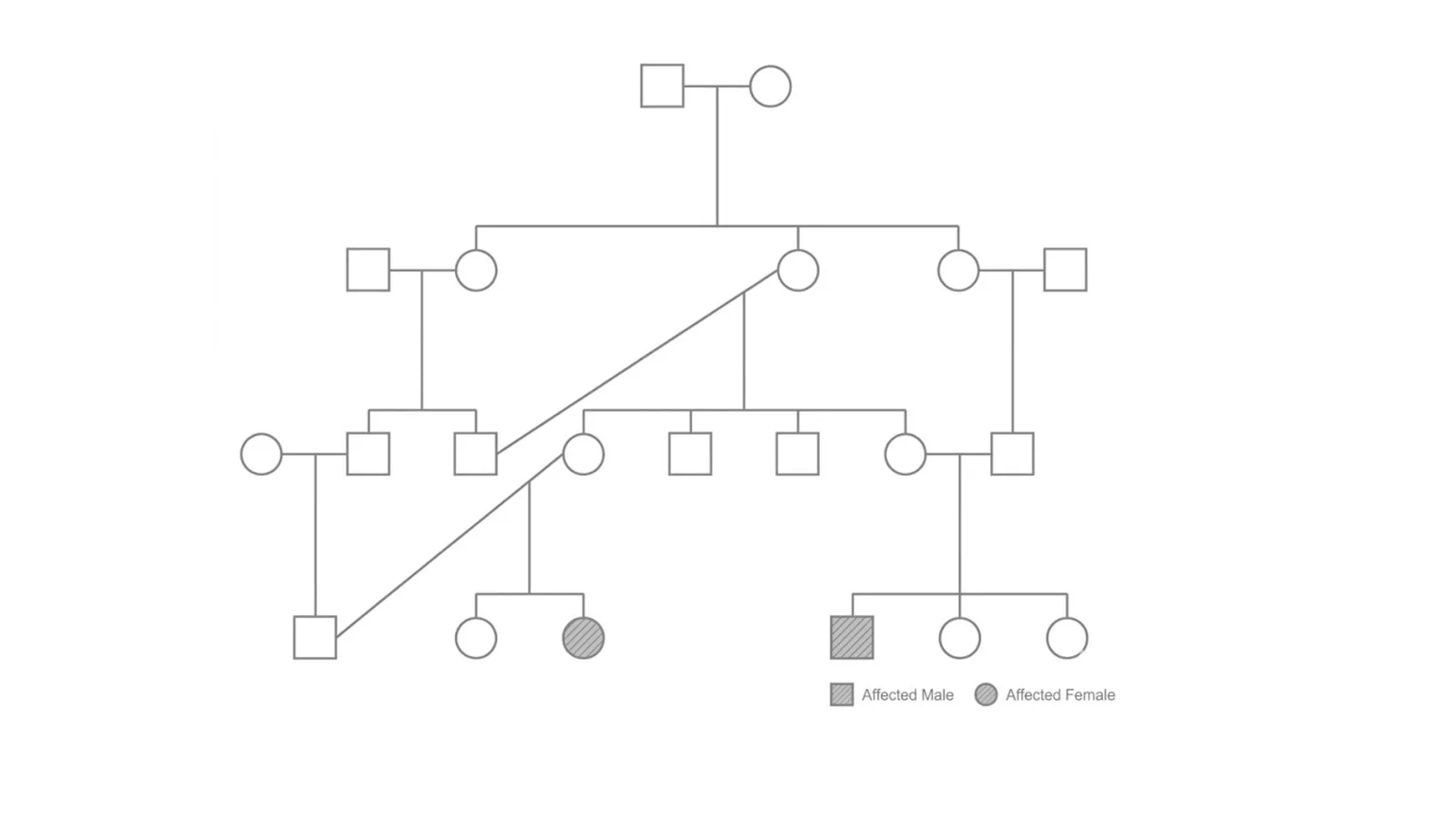
Multi-generational family pedigree of Case 1. Confirmed consanguinity is noted within parental lineages. The index female pediatric patient and her similarly affected male maternal cousin are indicated by the grey diagonal hatching, illustrating a classic autosomal recessive inheritance pattern.

Given a maternal cousin’s known history of familial chylomicronemia, targeted genetic testing by next-generation sequencing followed by Sanger sequencing confirmation was performed. This identified a homozygous deletion of exons 3-4 in the GPIHBP1 gene, confirming familial chylomicronemia syndrome type 1D (Figure 4).

**Figure 5:**
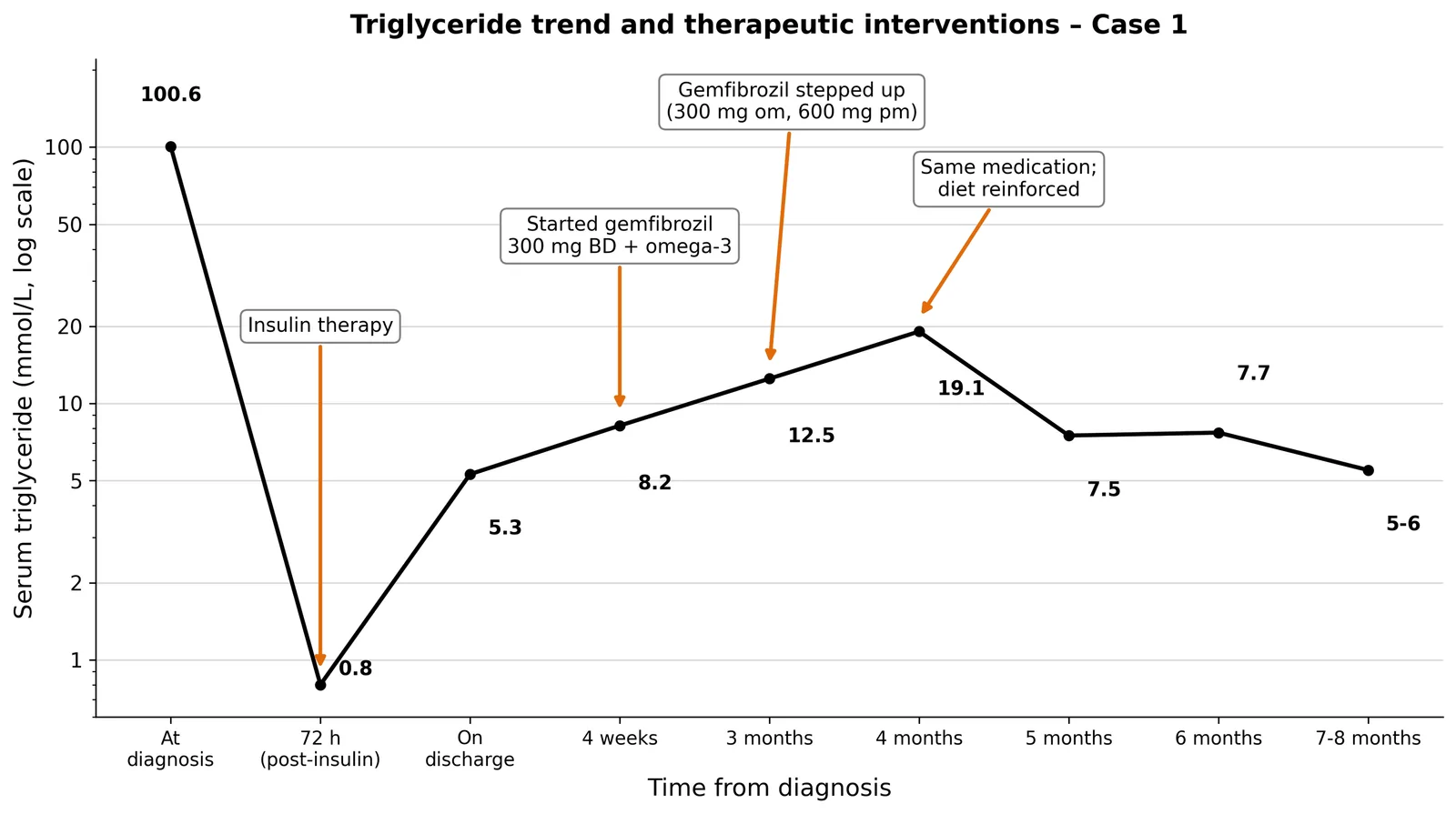
Serial serum triglyceride levels and corresponding interventions in Case 1

Post-discharge, initial dietary compliance was hindered by erratic pediatric eating behaviors and parental barriers regarding food composition, causing a rebound in triglycerides to 19.0 mmol/L. Despite intensive multidisciplinary counselling involving physicians, nurses, dietitians, and medical interpreters, triglyceride levels remained suboptimally controlled at approximately 8.0 mmol/L. Pharmacotherapy was therefore initiated with gemfibrozil when the patient was at 22 months of age in an attempt to optimise triglyceride level and prevent potential complications. The dosage of gemfibrozil was initiated at 300 mg twice daily (i.e., 50 mg/kg/day with the patient's body weight at 12 kg), and later up-titrated to 900 mg/day (300 mg in the morning, 600 mg in the evening, i.e., 75 mg/kg/day), supplemented with medium-chain triglyceride (MCT) oil and omega-3 fatty acids. Serum triglycerides subsequently stabilized between 5-6 mmol/L during follow-up (Figure 5). As fibrate therapy is not licensed in this age group, the off-label regimen was undertaken cautiously with structured safety monitoring. Liver function tests and creatine kinase were measured two weeks after initiation and monthly for the first three months, then every 2 to 3 months, which remained within normal limits throughout. The patient was also monitored clinically for gallbladder complications, with no reported abdominal pain and no evidence of cholelithiasis on follow-up.

Case 2

In contrast to the transport defect identified in Case 1, Case 2 illustrates a primary enzymatic deficiency. A full-term female neonate born to non-consanguineous parents presented on day 2 of life for neonatal jaundice. Visual inspection of routine blood samples incidentally revealed profound lipemia. A lipid panel confirmed hypertriglyceridemia with a triglyceride level at 10.6 mmol/L. A repeated fasting triglyceride level was also elevated at 8.5 mmol/L. Physical examination was unremarkable, with no eruptive xanthomas or dysmorphic features. Serum pancreatic enzymes and fundoscopy were normal.

Along the pipeline of diagnostic evaluation, her lipoprotein electrophoresis demonstrated a distinct chylomicron band and a dense very-low-density lipoprotein (VLDL) fraction. Given the exceptional presentation of neonatal hypertriglyceridaemia, next-generation sequencing was performed and identified two heterozygous missense variants in exon 6 of LPL (NM_000237.3): c.835C>G, p.(Leu279Val) and c.836T>G, p.(Leu279Arg), classified as likely pathogenic and confirmed by Sanger sequencing. Although the variants affect adjacent nucleotides within the same codon, parental testing demonstrated that they lie in trans: the father carried c.835C>G and the mother carried c.836T>G, with neither parent carrying both. This confirmed a diagnosis of compound heterozygous primary lipoprotein lipase deficiency.

Dietary management was immediately initiated by switching to a specialized formula containing 85% medium-chain triglycerides (MCT; Monogen). Serum triglycerides decreased to 6.0 mmol/L within one week, and she was safely discharged. Her investigation results are summarised in Tables 2-3.

**Table 2:** Lipid profile - Case 2 HDL: high-density lipoprotein; LPL: lipoprotein lipase; Monogen: a low-fat, MCT-based formula.

Investigation	At diagnosis	On discharge (after starting Monogen)	Reference range
Lipid profile			
Triglyceride	10.6 (random) --> 8.5 mmol/L (fasting)	6.0 mmol/L	< 0.8 mmol/L
Total cholesterol	4.8 mmol/L	3.9 mmol/L	< 4.4 mmol/L
HDL cholesterol	0.6 mmol/L	0.6 mmol/L	> 1.0 mmol/L

**Table 3:** Other laboratory findings – Case 2 LPL: lipoprotein lipase; VLDL: very-low-density lipoprotein.

Investigation	Finding
Lipoprotein assay	
Lipoprotein electrophoresis	Distinct chylomicron band with a dense very-low-density lipoprotein (VLDL) fraction
Genetic analysis	
LPL gene sequencing	Compound heterozygous LPL variants: NM_000237.2:c.835C>G and c.836T>G — confirming primary lipoprotein lipase deficiency
Familial screening	Both parents asymptomatic heterozygous carriers, consistent with autosomal recessive inheritance

**Figure 6:**
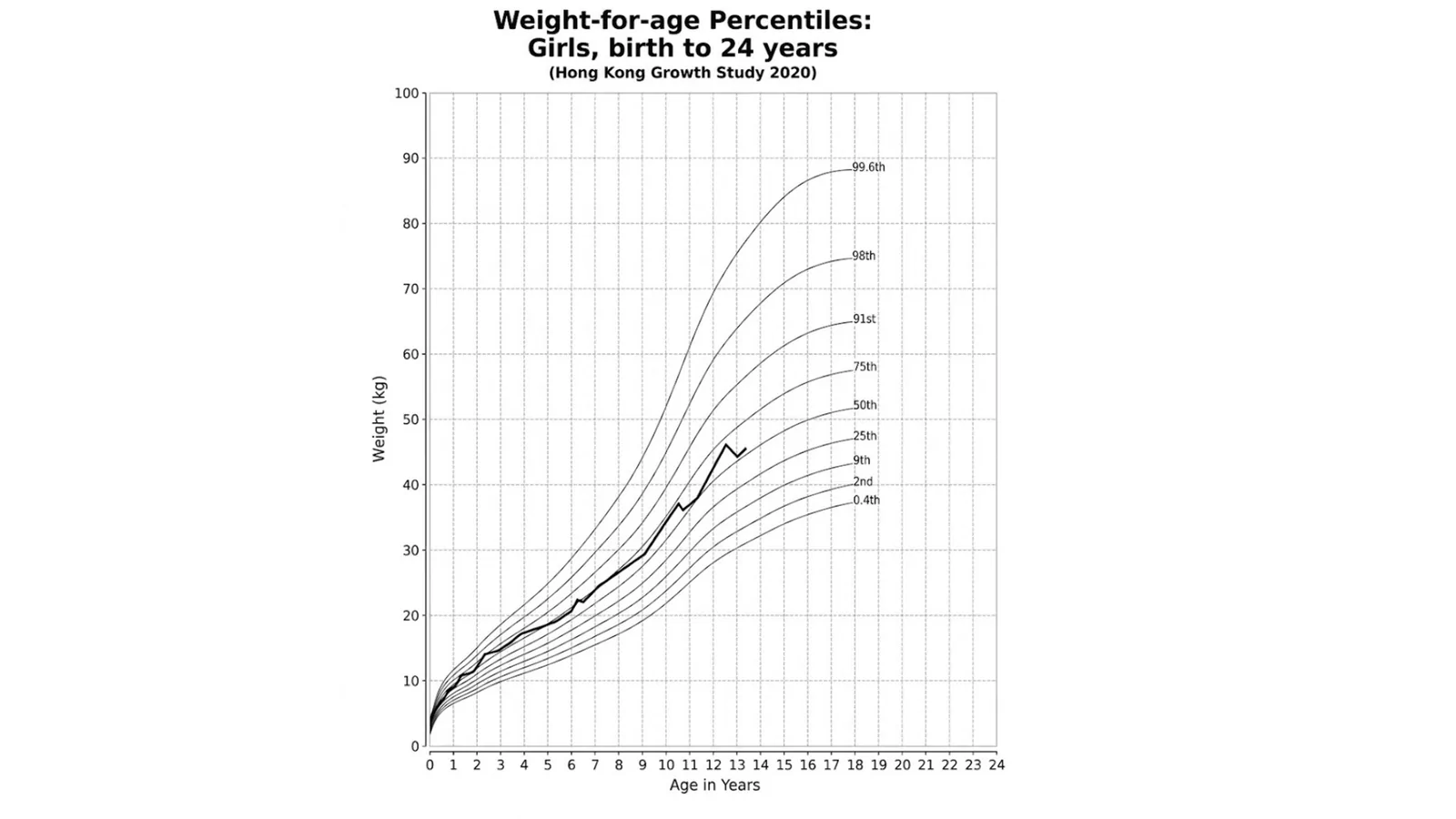
Weight-for-age percentiles of the female patient in Case 2 tracking from birth to age 13 years (Hong Kong Growth Study 2020). Weight-for-age percentiles demonstrate continuous, robust development progressing stably along the 75th percentile on restricted dietary fat modification [6].

Post-discharge, the girl remained clinically stable on dietary restriction alone without requiring conventional lipid-lowering pharmacotherapy. During adolescence, a transient rise in triglyceride to 5-6 mmol/L occurred, secondary to peer-related dietary non-compliance and diminished parental oversight. In 2023, adjunctive omega-3 fatty acid supplementation (4 g/day) was initiated. At her most recent evaluation at age 13, she was asymptomatic with an improved triglyceride level of 2.1 mmol/L. Throughout her longitudinal follow-up, development remained robust, with her weight-for-age percentile consistently tracking along the 75th percentile (Figure 6).

## Discussion

Monogenic familial chylomicronemia syndrome (FCS) is an ultra-rare metabolic disorder with an estimated global prevalence of 1-10 per million individuals, frequently obscured by under-recognition and clinical heterogeneity [7]. While certain founder populations exhibit higher clusters [8], data detailing pediatric cohorts within Asian populations, including Hong Kong, remain exceptionally sparse. This case series underscores the profound diagnostic pitfalls that contribute to delayed identification of this condition. Although the classic clinical triad of eruptive xanthomas, lipemia retinalis, and recurrent acute pancreatitis is well-established [9], early manifestations are frequently subtle, non-specific, or deceptive. Case 1 highlights the critical risk of diagnostic delay, where an atypical papulovesicular rash was initially mismanaged as an infectious process rather than being recognized as eruptive xanthomas. Conversely, Case 2 exemplifies the value of opportunistic screening, where the condition was revealed solely through visual inspection of a lipemic neonatal bilirubin sample. This stark contrast echoes an emerging consensus that pediatric FCS is consistently underdiagnosed due to the absent or atypical presentation of metabolic stigmata in early life.

Diagnosis

When pediatric FCS is clinically suspected, an immediate fasting lipid panel is essential to establish the diagnosis. A serum triglyceride-to-total-cholesterol ratio exceeding 10:1 (both calculated in mg/dL) serves as a robust biochemical signature pointing toward primary chylomicronemia [10,11]. Initial workup should rule out acute pancreatitis via serial pancreatic enzymes and selective abdominal imaging, evaluate for lipemia retinalis via fundoscopy, and systematically exclude secondary causes including hypothyroidism or type 1 diabetes mellitus. In resource-limited or pre-genetic settings, the North American Familial Chylomicronemia Score (NAFCS) may support the clinical identification of FCS [3]. By weighing criteria such as neonatal or infantile onset, severe hypertriglyceridemia, a low apolipoprotein B level, and the absence of secondary metabolic disruptors, an NAFCS score of ≥60 delivers 100% specificity and 66.7% sensitivity in distinguishing classical FCS from multifactorial chylomicronaemia [3]. However, the NAFCS was derived and validated exclusively in an adult lipid-clinic population and has not been validated in paediatric patients; it should therefore be applied with caution in young children.

While clinical and biochemical evaluation remains foundational to the diagnosis of FCS, molecular genetic analysis, where feasible, provides important adjunctive confirmation of the underlying aetiology and further informs management. Confirmation of causative variants within LPL, GPIHBP1, APOC2, APOA5, or LMF1 is mandatory to direct genotype-tailored care, establish precise long-term prognoses, and initiate cascade family screening and genetic counselling in regions with high consanguinity or strong family histories.

From a genetic perspective, our cases provide a direct comparison between two distinct defects within the lipoprotein lipase (LPL) pathway: Case 1, caused by a homozygous deletion in the GPIHBP1 gene, reflects impaired LPL transport; and Case 2, with compound heterozygous mutations in the LPL gene, represents a primary enzymatic deficiency. Emerging evidence suggests that classical LPL deficiency presents early with persistent hypertriglyceridaemia, while GPIHBP1 deficiency may exhibit greater phenotypic variability [12,13]. In contrast to this reported evidence, the patient in our case series with GPIHBP1-related disease seemed to exhibit more severe biochemical phenotype, presenting with a higher triglyceride level than the patient with LPL deficiency. This difference was also reflected in the treatment response: the GPIHBP1 patient required adjunctive fibrates in addition to dietary therapy, while the patient with LPL deficiency achieved sustained long-term biochemical stability with diet alone. This outcome suggests that genotype-phenotype correlations in paediatric FCS are not always substantiated. Disease severity is influenced not solely by the underlying genetic defect, but also by various confounding factors like the age and timing of diagnosis, dietary compliance and treatment intensity, etc. Further studies are required to better delineate genotype-phenotype correlations in paediatric FCS.

Treatment options

In FCS, defective lipoprotein lipase-mediated clearance leads to accumulation of chylomicrons, rendering triglyceride levels highly dependent on dietary fat intake. Dietary modification therefore remains the cornerstone of management. Current recommendations advocate a very low-fat diet, typically restricting fat intake to less than 10-15% of total daily energy, with supplementation using medium-chain triglycerides (MCTs), which bypass chylomicron formation through direct portal absorption [14]. To support optimal growth and prevent essential fatty acid deficiency, fats with high polyunsaturated fatty acid content such as walnut or grapeseed oil should be incorporated into the diet, which provide necessary omega-3 and omega-6 fatty acids within the restricted total fat allowance [4].

Dietary Therapy

In our case series, dietary therapy resulted in variable control of triglyceride levels, reflecting both the severity of the disease and the challenges associated with implementation. The patient in Case 1, who presented with severe hypertriglyceridemia due to GPIHBP1 deficiency, exhibited only partial control despite rigorous dietary counselling and adjunctive measures, with triglyceride levels fluctuating up to 8 mmol/L upon follow-up. Conversely, the patient in Case 2, diagnosed with lipoprotein lipase deficiency in the neonatal period, achieved sustained long-term control through dietary therapy alone, with triglyceride levels remaining well-managed into adolescence. This underscores that early diagnosis and prompt dietary intervention may lead to significant and sustained reduction in triglyceride level.

Maintaining long-term dietary adherence in pediatric hypertriglyceridemia presents significant challenges, shaped by age-specific developmental and psychosocial factors. In early childhood, as seen in Case 1, erratic eating patterns and evolving food preferences of toddlers complicate consistent dietary fat control. Furthermore, the rigorous fat restriction required necessitates a high level of health literacy and meticulous dietary calculation by caregivers. These efforts are often undermined by the cognitive burden of 'fat counting' and, in the present case, further exacerbated by language barriers. As patients transition into adolescence (Case 2), autonomy and peer influence often supersede parental oversight, leading to diminished dietary stringency. To mitigate these multifaceted barriers, a robust multidisciplinary framework comprising clinicians, specialized dietitians, and medical interpreters is essential to deliver culturally sensitive, longitudinal education and reinforced nutritional guidance.

Pharmacological Therapies

In the acute setting of severe hypertriglyceridemia, insulin infusion is effective in rapidly reducing triglyceride levels, as illustrated in Case 1, through upregulation of residual lipoprotein lipase activity. Of note, the role of insulin is limited in patients with minimal residual LPL activity. While plasmapheresis is considered an alternative to achieve rapid triglyceride reduction, evidence supporting its clinical benefit is limited, particularly in paediatric populations, and its use is generally reserved for severe or refractory cases [15]. The American Society for Apheresis classifies hypertriglyceridemia-induced pancreatitis as a Category III indication [16], reflecting the lack of definitive evidence and the need for individualised decision-making.

Pharmacological options for long-term management of paediatric familial chylomicronemia syndrome (FCS) remain limited, and no agents are currently specifically approved by the U.S. Food and Drug Administration (FDA) for this indication. This lack of formal paediatric approval means that standardized dosing guidelines are absent, requiring clinicians to rely on extrapolation from adult literature, case-by-case titration, and clinical response. Fibrates, such as gemfibrozil, act via activation of peroxisome proliferator-activated receptor-α and enhancement of lipolytic pathways; however, their efficacy is attenuated in FCS due to the underlying defect in lipoprotein lipase function [14]. Evidence in children is largely limited to case reports and small series. In a retrospective paediatric study evaluating dietary regimens in primary chylomicronemia, the addition of lipid-lowering agents including fibrates provided only modest additional triglyceride reduction beyond dietary intervention alone, with variable response between individuals [17]. Similarly, isolated paediatric case reports have described partial biochemical improvement with fibrates, although their overall effectiveness remains inconsistent and dependent on residual pathway activity [14]. In our cohort, gemfibrozil was introduced in Case 1 following suboptimal response to dietary therapy and resulted in modest additional triglyceride reduction without significant adverse effects, suggesting that fibrates may have a role as adjunctive therapy in selected patients. Nonetheless, careful counselling and monitoring are required given potential side effects, including hepatotoxicity, myopathy, and cholelithiasis.

Omega-3 fatty acids may also be used as adjunctive therapy, exerting triglyceride-lowering effects through reduction of hepatic very-low-density lipoprotein (VLDL) synthesis. In clinical practice, dosing is typically extrapolated from adult data, with regimens of up to 3-4 g/day of combined eicosapentaenoic acid (EPA) and docosahexaenoic acid (DHA) [18]. Paediatric data remain sparse, with small case series suggesting that omega-3 supplementation may provide incremental triglyceride reduction when combined with dietary therapy, although the magnitude of effect is modest and less pronounced in monogenic FCS [17,19]. In our patient, omega-3 supplementation was associated with biochemical stabilization rather than further significant reduction in triglyceride levels. Overall, these findings highlighted that while fibrates and omega-3 fatty acids may offer adjunctive benefit, dietary fat restriction remains the primary determinant of disease control, and pharmacological therapy should be individualised based on response and tolerability.

Novel Therapies

More recently, novel therapies targeting triglyceride metabolism independently of lipoprotein lipase activity have emerged. Inhibition of apolipoprotein C-III (APOC3) has shown particular promise: volanesorsen achieves substantial triglyceride reduction but is limited by adverse effects, notably thrombocytopenia [20,21]. Olezarsen, a next-generation APOC3 antisense oligonucleotide, received FDA approval in December 2024 as an adjunct to diet for adults with FCS, and plozasiran, a small interfering RNA targeting APOC3, was FDA-approved for the same indication in November 2025; both demonstrated substantial triglyceride lowering with more favourable safety profiles than volanesorsen [21-23]. Therapies targeting angiopoietin-like protein 3 (ANGPTL3) are also under investigation [23]. However, neither olezarsen nor plozasiran is currently approved for paediatric use, and experience with these agents in children remains very limited. Long-term safety, efficacy, and accessibility of these novel agents in this age group are yet to be established.

## Conclusions

This two-case series has highlighted the heterogeneity of clinical presentation and treatment response in paediatric FCS. Early diagnosis and prompt initiation of appropriate treatment are important in FCS, as untreated or poorly controlled disease carries a substantial risk of serious complications such as acute pancreatitis. Strict dietary intervention is critical in achieving optimal outcomes, although real-world factors including dietary adherence and socioeconomic constraints may limit its effectiveness. Pharmacological therapies such as fibrates and omega-3 fatty acids provide only modest additional benefits. While emerging targeted therapies offer promising future options, their role in paediatric practice remains to be established. A multidisciplinary approach with individualised management is therefore essential to optimise long-term outcomes in this rare but potentially life-threatening condition. Given that pharmacological therapies like fibrates and omega-3 fatty acids are used off-label, lacking international consensus guidelines and standardised paediatric dosing, needs exist for long-term efficacy data and formal studies to establish safe and effective treatment regimens in paediatric FCS.
